# Pharmaceutical registration, essential medicines, and registration depth in Sri Lanka: a longitudinal analysis, 1991–2025

**DOI:** 10.1080/20523211.2026.2735527

**Published:** 2026-09-25

**Authors:** Manori Jayasinghe, Amanda Rush, Andrew Wilson

**Affiliations:** a Faculty of Medicine and Health, School of Public Health, Leeder Centre for Health Policy, Economics and Data, The University of Sydney, Newtown, NSW, Australia; b Department of Pharmacy, Faculty of Allied Health Sciences, The University of Ruhuna, Galle, Sri Lanka

**Keywords:** Pharmaceutical registration, essential medicines, market structure, low- and middle-income countries

## Abstract

Pharmaceutical registration determines which medicines may enter the market, yet its role in shaping pharmaceutical market structure in relation to essential medicines has received limited attention, particularly in low- and middle-income countries. This study examined how pharmaceutical registration portfolios in Sri Lanka evolved in relation to essential medicines alignment, therapeutic composition, and sources of supply. Pharmaceutical registrations were analysed in Sri Lanka at three time points (1991, 2009, and 2025) to examine changes in the structure of the registration portfolio and alignment with the Essential Medicines List (EML) at both product and active substance levels. Data for 1991 were derived from a published study, while analyses were conducted for 2009 and 2025 using national registration records. The number of registered products increased substantially between 1991 and 2009 and remained high in 2025, while the number of active substances changed modestly. Product-level alignment with the EML declined from 71.0% in 1991 to 38.0% in 2025, whereas active-substance-level alignment increased from 31.4% to 59.4%. This divergence reflected broader coverage of essential active substances alongside fewer products per substance. The composition of the register shifted, with a declining share of anti-infective medicines and increasing representation of medicines for chronic conditions. The registration portfolio remained import dependent, although the proportion of locally manufactured substances increased between 2009 and 2025. Registration depth among active substances was uneven, with many substances represented by few or no registered products. Pharmaceutical registration in Sri Lanka remained broadly aligned with essential medicines priorities over time, but this alignment occurred through changing portfolio structures. The study shows that assessments based on the number of registered products may not capture therapeutic breadth at the active-substance level. The findings highlight the importance of considering registration depth and import dependence when interpreting pharmaceutical market structure and supply vulnerabilities in import-dependent health systems.

## Introduction

Access to essential medicines in low- and middle-income countries (LMICs) is influenced by pricing, procurement, and prescribing practices (Hogerzeil et al., 1993; Laing et al., 2003; Management Sciences for Health & World Health Organization, 1997; Peacocke et al., 2022), but is also shaped by upstream regulatory decisions governing pharmaceutical market entry (World Health Organization, 2019). Pharmaceutical registration systems define which products may legally enter and remain within the formal pharmaceutical market and therefore influence the structure and therapeutic composition of registered medicine portfolios over time (World Health Organization, 2019). However, few empirical studies have examined how pharmaceutical registration portfolios correspond with essential medicines priorities over time, particularly in LMICs.

In many LMICs, pharmaceutical markets are heavily dependent on imported medicines supplied through transnational manufacturing and distribution networks, increasing the regulatory importance of maintaining coherent and resilient registration systems. The essential medicines concept, developed by the World Health Organization (WHO), provides a framework for rational medicines selection based on evidence and population health needs. Extensive literature has examined the adoption of national essential medicines lists (EML), their role in public sector procurement, and their influence on prescribing behaviour (Hogerzeil et al., 1993; Laing et al., 2003; Management Sciences for Health & World Health Organization, 1997; Peacocke et al., 2022). Far less attention has been given to how pharmaceutical registration portfolios correspond with essential medicines priorities over time. In many LMICs, registration decisions are formally based on assessments of quality, safety, and efficacy, with limited explicit consideration of therapeutic duplication, portfolio concentration, or broader implications for pharmaceutical market structure. Whether registration portfolios nonetheless evolve in ways broadly consistent with essential medicines priorities remains unclear.

Sri Lanka adopted essential medicines principles early (Bibile, 1977), established a national formulary for the public sector (Lall & Bibile, 1977), and introduced mandatory pharmaceutical registration in the late 1980s (Attanayake & Siyambalagoda, 2003). In a landmark analysis published in the Bulletin of the World Health Organization in 1993, Weerasuriya showed that more than 70% of registered pharmaceutical products corresponded to medicines (or alternatives) listed in the EML, despite the list being applied to the public sector and registration being a universal requirement for market entry (Weerasuriya, 1993). This finding suggested that essential medicines principles influenced market composition indirectly through regulatory and professional norms, even without explicit mandates at the point of registration.

Since that assessment, Sri Lanka’s pharmaceutical system has changed substantially. The number of registered products increased across the study period (Department of Pharmacology, University of Colombo, 2009; National Medicines Regulatory Authority, 2025; Weerasuriya, 1993), patterns of disease shifted toward chronic non-communicable conditions (Ediriweera et al., 2018), private-sector medicine supply remained substantial (Amarasinghe et al., 2021), and institutional reforms reshaped the regulatory environment (Government of Sri Lanka, 2015). These changes raise questions that could not be addressed in the earlier study. It remains unclear whether alignment with essential medicines reflects expansion of therapeutic coverage at the level of active substances or proliferation of product variants around selected active substances; whether shifts in therapeutic composition correspond with evolving health needs; and how registration portfolios evolved under changing regulatory and market conditions.

This study analyses pharmaceutical product registrations in Sri Lanka at three time points: 1991, 2009, and 2025. By distinguishing between registered pharmaceutical products and active substances, the analysis examines how alignment with the EML, therapeutic composition, and registration depth evolved over time within the formal pharmaceutical market. The study further examines changes in sources of supply within an import-dependent pharmaceutical system. Rather than assessing medicine utilisation, procurement behaviour, or patient access, the analysis focuses on registration portfolios as regulatory outputs that define the structure and composition of the registered pharmaceutical market.

## Methods

A comparative cross-sectional analysis was conducted of pharmaceutical product registrations in Sri Lanka at three time points: 1991, 2009, and 2025. The analysis examined changes in the size and structure of the pharmaceutical register, alignment with the EML, therapeutic composition, and sources of supply. Pharmaceutical registration was conceptualised as a regulatory output defining the formal scope of the pharmaceutical market. The study did not assess medicine utilisation, availability, procurement performance, or prices.

### Data sources, study periods and comparability

Data for 1991 were obtained from Weerasuriya (1993), which analysed all product registrations recorded up to June 1991. Data for 2009 were obtained from the Sri Lanka Drug Index 2009–2010 (Department of Pharmacology, University of Colombo, 2009). Data for 2025 were obtained from the registration dataset published by the National Medicines Regulatory Authority (NMRA), dated 31 July 2025 (National Medicines Regulatory Authority, 2025). The datasets represent national product registration registers at defined time points. Although obtained from different sources, all data originated from Sri Lanka’s national medicines regulatory records.

Consistent analytical approaches were applied across datasets to support comparability across study periods. The primary unit of analysis was the registered pharmaceutical product, defined as a specific active substance or combination of substances in a given dosage form and strength, recorded as a distinct regulatory entry. A secondary unit of analysis was the unique active substance, defined using the International Nonproprietary Name. The term active substance is used throughout the manuscript to refer to unique active substances.

Records with incomplete product information were excluded only where the missing information prevented classification for the relevant analysis. Examples included records where the active substance or product composition could not be reliably identified for active-substance-level analysis, or where the available information was insufficient to assign the product to a first-level ATC category. For 1991, this reflects exclusions reported in the original published analysis (31 registrations). For the 2009 analysis, four active substances were excluded due to incomplete information. For the ATC analysis, 809 combination products and 39 registrations with insufficient classification information were excluded, leaving 5,998 registrations. For the 2025 dataset, 232 registrations were excluded from ATC analyses, comprising 219 combination products and 13 records with incomplete therapeutic classification. Combination products were not treated as incomplete records; they were retained as distinct product entries but excluded from active-substance-level analyses.

Although the 1991 analysis distinguished between over-the-counter and prescription-only products, this categorisation was not applied to the 2009 and 2025 datasets because definitions and recording practices were not consistent across time and data sources, limiting its suitability for longitudinal comparison. Dispensing status was not used to derive product counts, active-substance counts, EML alignment, therapeutic composition, or sources-of-supply analyses in any study year, and its exclusion therefore does not affect the reported results.

Country of manufacture was defined as the manufacturing country recorded for each registered product in the relevant registration source. It did not refer to the registered office of the company or the country from which the product was imported, unless that country was recorded as the manufacturing country. Products recorded as manufactured in Sri Lanka were classified as domestic, and products recorded with a non-Sri Lankan manufacturing country were classified as imported.

### Essential medicines list alignment

For 1991, EML alignment was taken directly from Weerasuriya (1993), who reported alignment based on the national Essential Drugs List 1988. The wording ‘EML-listed medicines or specified alternatives' reflects the terminology used in Weerasuriya’s published analysis. For 2009 and 2025, alignment was assessed against medicines listed in the relevant national EMLs published in 2009 and 2013–2014 respectively (Expert Committee on Essential Medicines, 2009; Ministry of Health, 2014), including core and complementary sections. Results were reported as overall EML alignment for each study year. Although Sri Lanka issued an EML in 2022 in response to acute supply disruptions during the economic crisis (Ministry of Health, 2022), this list functioned as a selective, crisis-driven prioritisation tool rather than a comprehensive national revision and was therefore not used for alignment analysis.

### Therapeutic classification

Therapeutic composition was assessed using the WHO/ATC classification system. For the 2009 dataset, ATC categories were taken directly from the Sri Lanka Drug Index. For the 2025 dataset, ATC coding at the first anatomical level was applied using the WHO ATC/DDD Index 2025 (World Health Organization Collaborating Centre for Drug Statistics Methodology, 2025). Analyses were restricted to the first anatomical level to support comparability across time points and reduce inconsistencies arising from differences in coding sources as described above.

### Data analysis

Descriptive analyses were conducted for each study year and compared across time points. The analysis distinguished between therapeutic breadth, registration depth, and portfolio saturation. Therapeutic breadth referred to the range of active substances represented in the register; in the EML analysis, it referred specifically to the number or proportion of EML-listed active substances represented. Registration depth was defined as the number of registered products corresponding to a given active substance. Portfolio saturation was defined as the ratio of registered products to active substances across the whole register, representing the average number of registered products per active substance. In contrast, registration depth describes how product registrations are distributed across individual active substances.

EML alignment was summarised as the proportion of registered products and active substances aligned with the list for each study year. Comparing product-level and active-substance-level alignment helped distinguish between concentration of multiple product variants around selected medicines and wider representation of essential active substances.

Therapeutic composition was summarised as proportional distributions across ATC categories and presented using tables and graphical displays. All analyses were conducted using Microsoft Excel.

## Results

### Evolution of pharmaceutical registrations, 1991–2025

The total number of registered products increased from 3,405 in 1991 to 6,846 in 2009, before declining to 6,070 in 2025 (Figure 1). Over the same period, the number of active substances showed more limited variation, with 773 active substances registered in 1991, 684 in 2009, and 729 in 2025. The ratio of registered products to active substances increased substantially between 1991 and 2009, rising from approximately 4.4 to10.0 products per active substance, and remained elevated in 2025 at 8.3 products per active substance (Figure 1).

**Figure 1.**
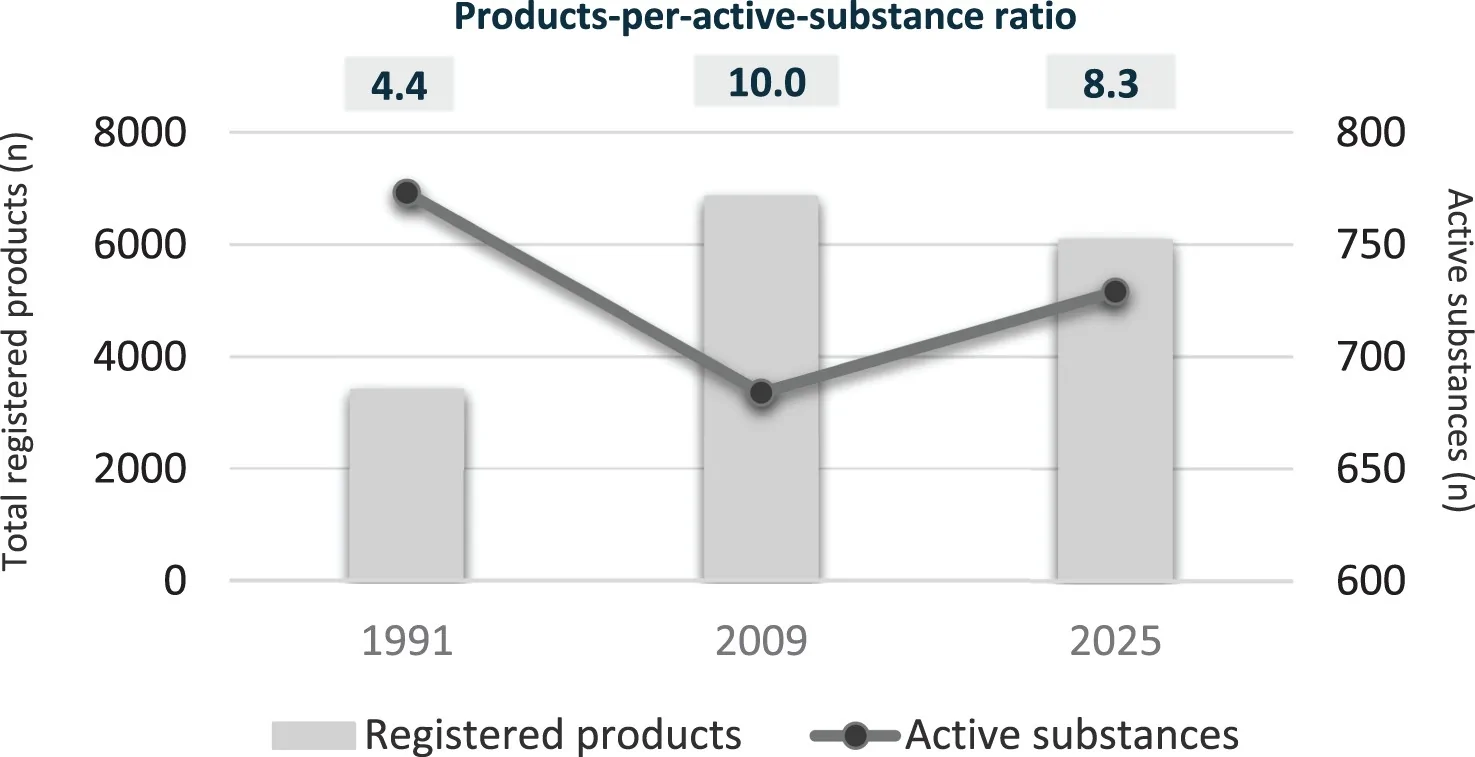
Trends in pharmaceutical registrations in Sri Lanka, 1991–2025.

Bars indicate the total number of registered products. The line indicates the number of active substances. Values displayed at the top of the figure indicate the number of products per active substance, reflecting changes in the ratio of products to active substances over time.

### Alignment with the essential medicines list

Alignment with the EML was assessed at both product and active-substance levels for each study year (Table 1; Figure 2). At the product level, 2,429 of 3,405 registered products in 1991 were aligned with the EML or specified alternatives (71.0%). This proportion declined to 62.5% in 2009 (4,276 of 6,846 registrations) and further to 38.0% in 2025 (2,306 of 6,070 registrations). At the active-substance level, 31.4% of registered substances were aligned with the EML in 1991. Alignment increased to 50.0% in 2009 and to 59.4% in 2025.

**Figure 2.**
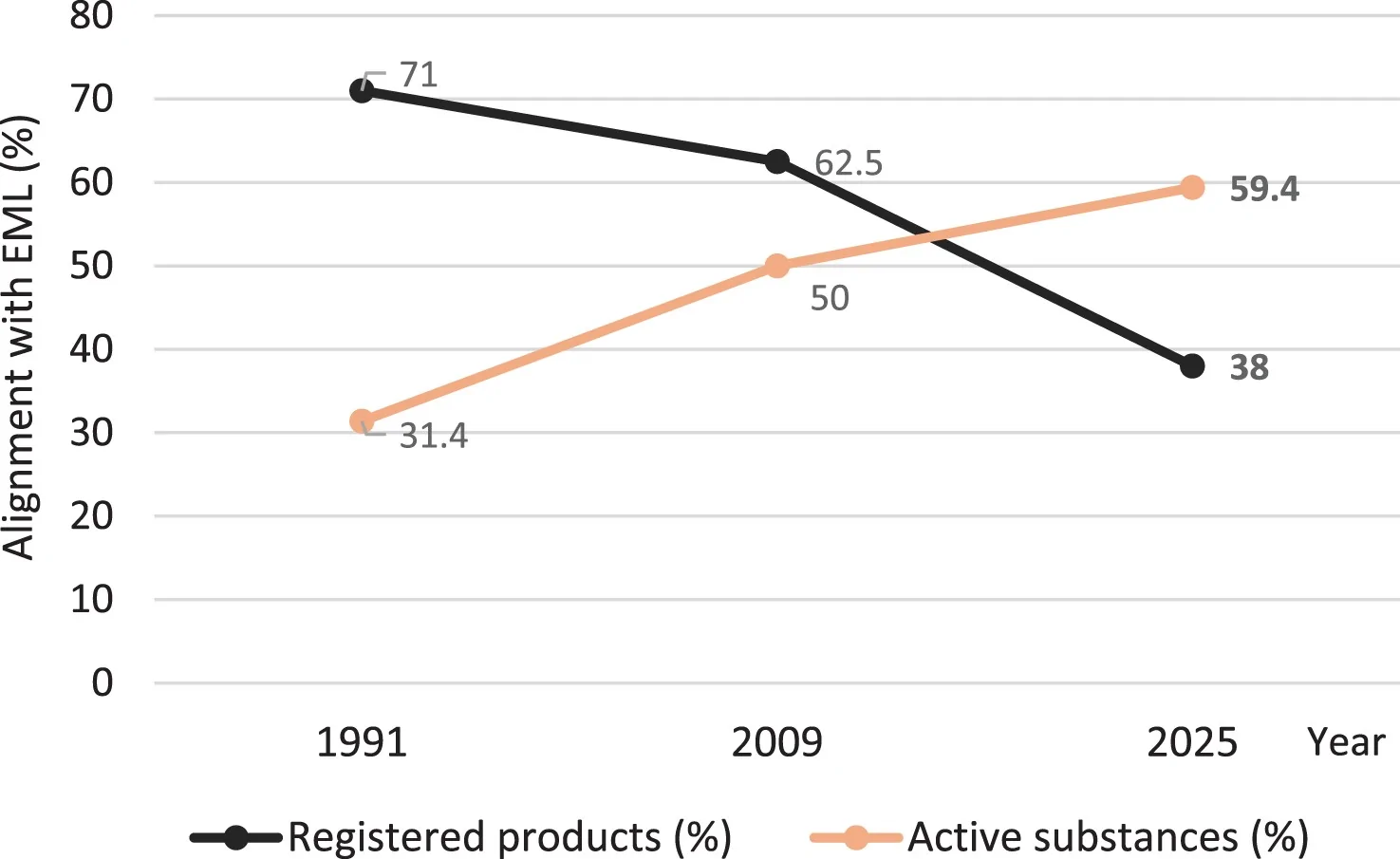
Diverging trends in product-level and active-substance-level alignment with the Essential Medicines List, Sri Lanka, 1991–2025.

**Table 1.** Alignment of registered pharmaceutical products and active substances with the national Essential Medicines List (EML), 1991–2025.

Year	Registered products (n)	Products aligned with EML (n,%)	Active substances (n)	Active substances aligned with EML (n,%)
1991	3,405	2,429 (71.0%)	773	243 (31.4%)
2009	6,846	4,276 (62.5%)	684	342 (50.0%)
2025	6,070	2,306 (38.0%)	729	433 (59.4%)

Note: The 1991 percentage is reproduced from the original source (71%); recalculation from the reported counts gives 71.3%.

The relationship between product-level and substance-level EML alignment shifted over time. In 1991 and 2009, alignment was greater at the product level, but by 2025 the pattern had reversed.

Percentages represent the proportion of registered products and active substances aligned with EML in each study year. Product-level alignment declined over time, while alignment at the level of active substances increased.

In 2025, the number of registered products per active substance among medicines listed in the 2013/2014 EML was unevenly distributed (Figure 3). Of the 590 listed substances of the EML, 157 had no registered products and 85 were represented by a single product. A further 180 substances had two to four products and 116 had five to nine products, while only 38 substances had 10–20 registered products and 14 had 21 or more.

**Figure 3.**
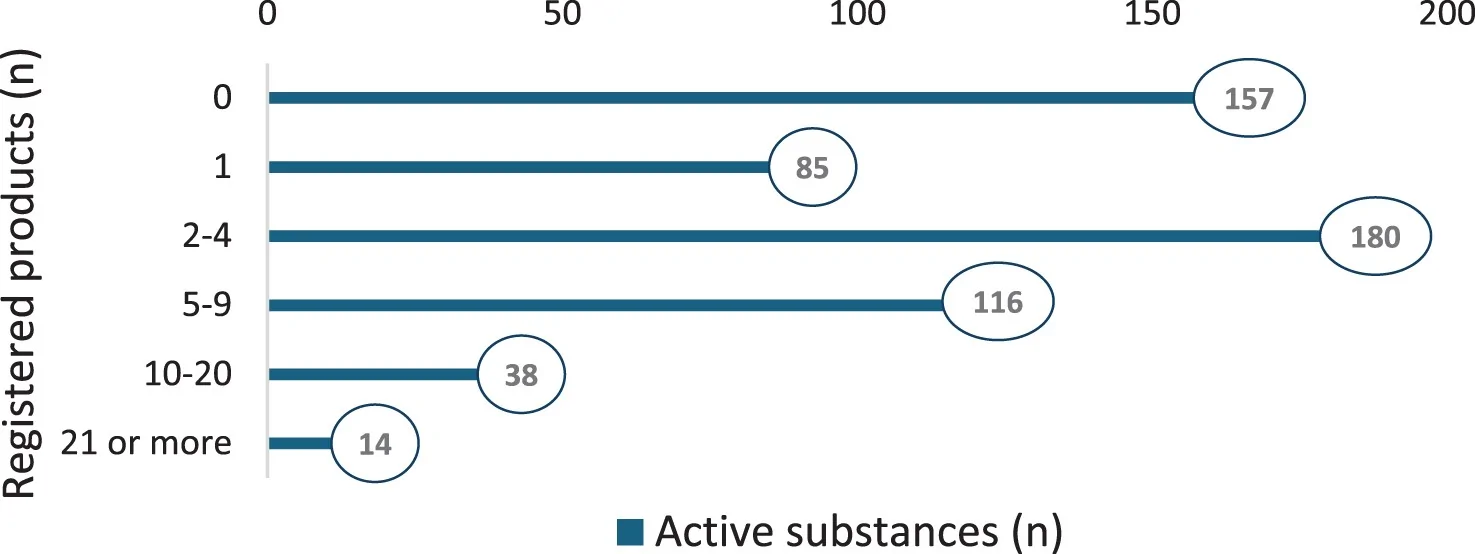
Uneven registration depth among EML-listed active substances in Sri Lanka, 2025.

Bars show the number of EML-listed active substances (*n*  =  590) according to the number of registered products in 2025, illustrating substantial variation in registration depth.

### Therapeutic composition of registered products

The therapeutic composition of registered products changed over the study period (Table 2; Figure 4). In 1991, anti-infectives constituted the largest category (17.0%), followed by alimentary tract and metabolism medicines (15.0%). Nervous system, respiratory system, and dermatological products each accounted for about 11.0%, while cardiovascular medicines represented 8.0% of registrations.

**Figure 4.**
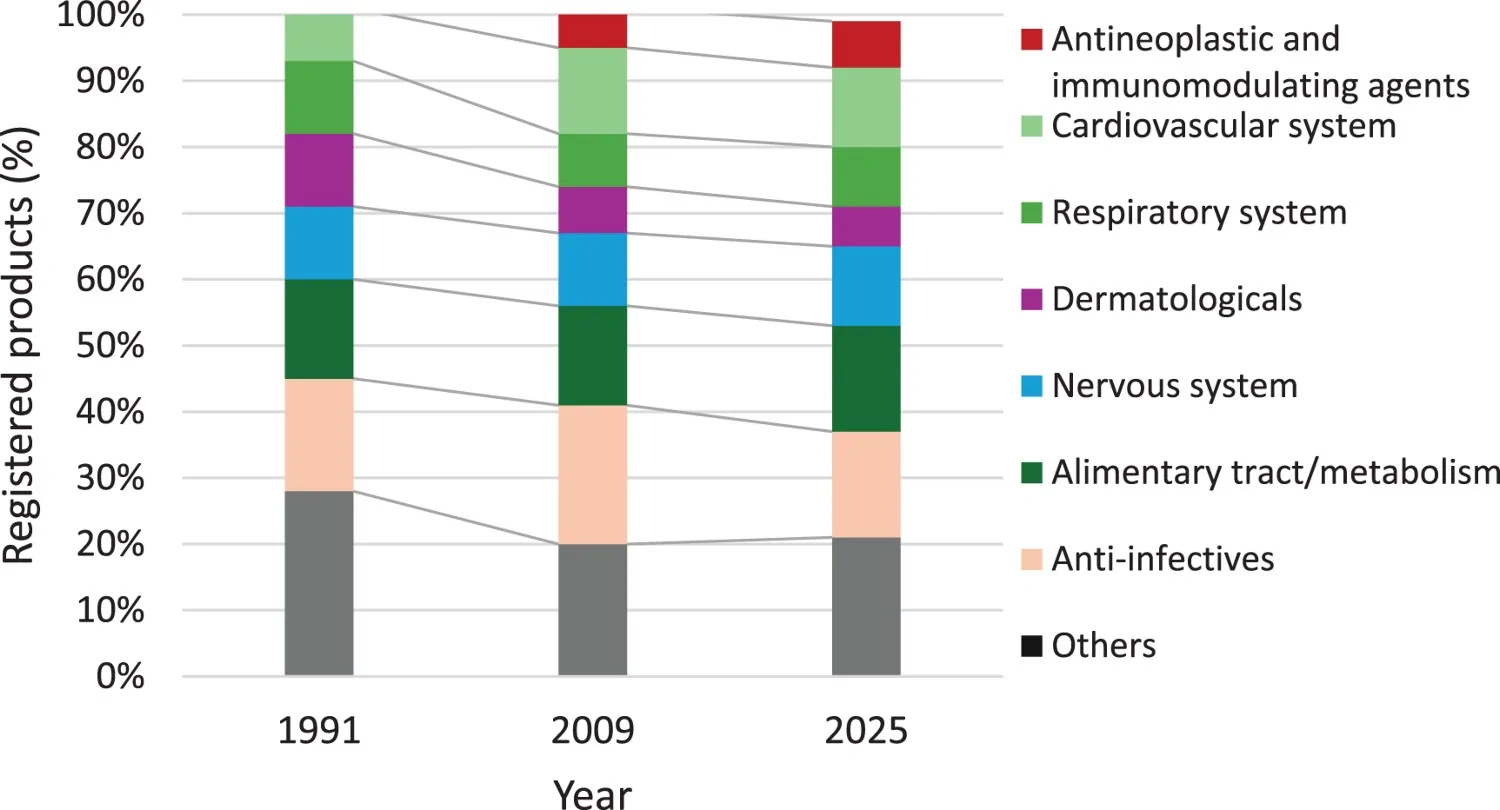
Distribution of registered pharmaceutical products by World Health Organization Anatomical Therapeutic Chemical (ATC) classification, 1991–2025.

**Table 2.** Distribution of registered products by ATC category.

ATC Category	1991 (n, %)	2009* (n, %)	2025** (n, %)
Anti-infectives	562 (17.0%)	1,237 (21.0%)	910 (16.0%)
Alimentary tract/metabolism	518 (15.0%)	904 (15.0%)	956 (16.0%)
Nervous system	379 (11.0%)	644 (11.0%)	715 (12.0%)
Respiratory system	360 (11.0%)	454 (8.0%)	512 (9.0%)
Dermatologicals	362 (11.0%)	412 (7.0%)	374 (6.0%)
Cardiovascular system	290 (8.0%)	773 (13.0%)	695 (12.0%)
Antineoplastic and immunomodulating agents	#	367 (6.0%)	425 (7.0%)
Other	934 (28.0%)	1,207 (20.0%)	1,251 (21.0%)

# The number of registrations for antineoplastic and immunomodulating agents was not reported separately in Weerasuriya (1993).

*2009 values are based on 5,998 product registrations. Combination products (*n* = 809) and records that could not be assigned to a specific ATC category (*n* = 39) are not included in the values shown in this table.

**2025 values are based on 5,838 product registrations. Combination products (*n* = 219) and records with incomplete therapeutic classification (*n* = 13) are not included in the values shown in this table. Percentages are calculated as proportions of included products and rounded to whole numbers; totals may not sum to 100% due to rounding.

In 2009, anti-infectives remained the largest category (21.0%). The share of cardiovascular medicines increased to 13.0%, while alimentary tract and metabolism and nervous system categories accounted for 15.0% and 11.0%, respectively. Respiratory system and dermatological products declined to 8.0% and 7.0%, and antineoplastic and immunomodulating agents accounted for 6.0%.

By 2025, alimentary tract and metabolism medicines and anti-infectives each accounted for 16.0% of registrations, followed by nervous system and cardiovascular medicines (12.0% each). Dermatologicals decreased further to 6.0%. Antineoplastic and immunomodulating agents increased to 7.0%. The proportion of products grouped under ‘other' categories declined from 28.0% in 1991 to 20.0% in 2009 and increased slightly to 21.0% in 2025.

Bars represent the percentage distribution of registered products across ATC categories in each study year. Percentages are calculated as proportions of classified products within each year. Changes over time reflect shifts in the relative distribution of registered products across ATC categories.

### Concentration among frequently registered active substances

The most frequently registered active substances varied across study years, with limited overlap in the leading active substances (Table 3).

**Table 3.** Most frequently registered active substances in 1991, 2009, and 2025.

Active substance	1991 (n)	2009 (n)	2025 (n)
Ampicillin	75	–	–
Amoxycillin	73	99	85
Cotrimoxazole	68	–	–
Paracetamol	67	62	59
Salbutamol	45	61	–
Metronidazole	42	57	–
Hydrocortisone	42	–	–
Neomycin	40	–	–
Dexamethasone	38	–	–
Betamethasone	33	–	–
Atorvastatin	#	101	80
Cefuroxime	#	99	74
Ciprofloxacin	#	96	–
Losartan	#	84	66
Glimepiride	#	77	–
Azithromycin	#	75	75
Cefalexin	#	74	–
Metformin	#	73	73
Omeprazole	#	63	–
Esomeprazole	#	52	89
Rosuvastatin	#	–	89
Diclofenac	#	–	84
Gliclazide	#	–	63

# Values were not reported separately in Weerasuriya (1993).

– Indicates that the active substance was not among the most frequently registered active substances in the particular year, even though registrations may still exist.

In 1991, commonly registered active substances were predominantly anti-infectives, including ampicillin (*n* = 75), amoxycillin (*n* = 73), and cotrimoxazole (*n* = 68), along with respiratory and corticosteroid agents such as salbutamol (*n* = 45) and hydrocortisone (*n* = 42).

By 2009, the most frequently registered active substances included atorvastatin (*n* = 101), cefuroxime (*n* = 99), and ciprofloxacin (*n* = 96), reflecting a broader range of therapeutic classes. Several active substances, including amoxycillin (*n* = 99) and azithromycin (*n* = 75), remained prominent.

In 2025, frequently registered active substances included esomeprazole and rosuvastatin (*n* = 89 each), diclofenac (*n* = 84), and atorvastatin (*n* = 80). Some active substances, such as amoxycillin (*n* = 85), azithromycin (*n* = 75), and metformin (*n* = 73), appeared consistently across multiple years.

### Sources of supply and country of manufacture

The country of manufacture of registered products shifted substantially over time (Table 4; Figure 5). In 1991, imports accounted for 91.0% of registered products, with domestic manufacture in Sri Lanka comprising 9.0%. By 2009, the domestic share had declined to 2.8%, while imports accounted for 97.2% of registrations, driven largely by products manufactured in India (40.6%) and Pakistan (15.7%). In 2025, the domestic share increased to 13.4%, alongside continued growth in products manufactured in India (52.3%), while the aggregate share of products manufactured in other foreign countries declined.

**Figure 5.**
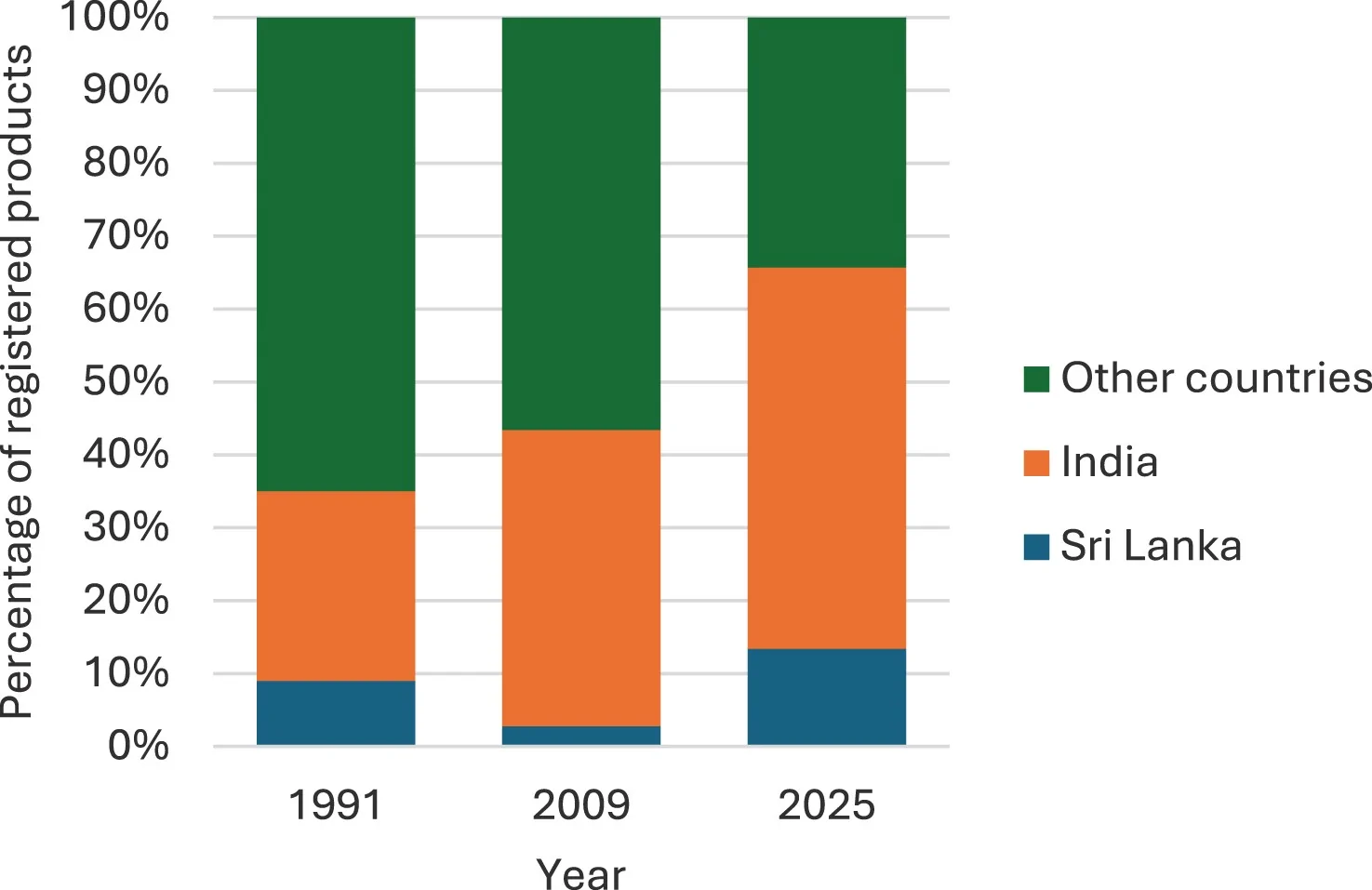
Distribution of registered pharmaceutical products by country of manufacture, 1991–2025.

**Table 4.** Country of manufacture and domestic share of registered products.

Country of manufacture	1991 (n, %)	2009 (n, %)	2025 (n, %)
India	886 (26.0%)	2,778 (40.6%)	3,176 (52.3%)
Sri Lanka	305 (9.0%)	192 (2.8%)	815 (13.4%)
Pakistan	158 (5.0%)	1,072 (15.7%)	587 (9.7%)
Bangladesh	*	112 (1.6%)	583 (9.6%)
United Kingdom (UK)	469 (14.0%)	685 (10.0%)	41 (0.7%)
United States of America (USA)	146 (4.0%)	321 (4.7%)	32 (0.5%)
Switzerland	199 (6.0%)	218 (3.2%)	84 (1.4%)
Germany	156 (5.0%)	212 (3.1%)	64 (1.1%)
Malaysia	*	16 (0.2%)	100 (1.6%)
Thailand	*	202 (3.0%)	46 (0.8%)
France	*	251 (3.7%)	54 (0.9%)
Other Countries	1,086 (31.0%)	787 (11.5%)	488 (8.0%)
Total	3,405 (100%)	6,846 (100%)	6,070 (100%)

* Values were not reported separately in Weerasuriya (1993).

Bars show observed percentages for each study year. ‘Other foreign countries’ includes all non-Sri Lankan countries of manufacture other than India. Percentages represent the share of total registered products in each year.

## Discussion

This study shows that pharmaceutical registration in Sri Lanka remained broadly aligned with essential medicines priorities over three decades, but through changing portfolio structures. While product-level alignment with the EML declined over time, alignment at the level of active substances increased substantially, indicating broader therapeutic coverage alongside lower registration density per substance. These findings highlight the importance of distinguishing between registered products and active substances when interpreting pharmaceutical market structure.

### Depth versus breadth of essential medicines alignment

A key contribution of this study is the distinction between depth and breadth of essential medicines alignment. Higher product-level than active-substance-level alignment in 1991 and, to a lesser extent, 2009 indicates that registrations remained concentrated within selected essential active substances, with multiple brands, dosage forms, or strengths registered for the same substances. However, the 2009 estimate also shows that active-substance-level EML coverage had already expanded substantially by that time.

By 2025, the pattern had reversed, with active-substance-level alignment exceeding product-level alignment. This indicates wider formal coverage of essential active substances in the register, despite fewer corresponding product registrations. This inversion demonstrates that similar levels of EML alignment can arise through different portfolio structures, with distinct implications for market organisation and regulatory oversight.

Across the study period, the number of registered products increased substantially, while the number of active substances changed relatively little. This disproportionate growth helps explain the changing relationship between product-level and active-substance-level alignment, with registrations becoming increasingly concentrated around a relatively stable set of therapeutic substances (Kaplan et al., 2013). The resulting increase in products per active substance points to increased saturation of the registration portfolio, most evident in 2009. In low- and middle-income settings, this pattern underscores the importance of assessing not only the volume of registrations but their distribution across active substances when evaluating alignment with essential medicines priorities.

The number of registered active substances declined between 1991 and 2009 and showed a partial recovery by 2025, but did not return to the level observed in 1991. This pattern reflects net changes in the composition of the registration portfolio over time. Available registration records do not allow disaggregation of whether these changes resulted from deregistration of products no longer actively maintained on the register, changes in classification practices or harmonisation of International Nonproprietary Names, or other routine processes of register maintenance. This pattern indicates that pharmaceutical registers evolve through both entry and exit rather than acting as cumulative inventories.

### Shifts in therapeutic composition

The observed changes in therapeutic composition suggest a gradual reorientation of the pharmaceutical register toward medicines used for long-term management of non-communicable diseases. While anti-infective medicines remained prominent, their relative importance declined over time as registrations increasingly reflected demand for chronic disease therapies. Antineoplastic and immunomodulating agents were reported separately in both 2009 and 2025, with their share increasing from 6.0% to 7.0%. These patterns are consistent with Sri Lanka’s epidemiological transition toward non-communicable diseases and longer-term treatment needs (Ediriweera et al., 2018). Although this study does not assess causality, the findings suggest that registration portfolios evolve in response to sustained patterns of clinical demand and market opportunity, rather than remaining aligned with the historical disease profile.

### Market composition and sources of supply

Changes in the structure of the registration portfolio must be interpreted in the context of a pharmaceutical market that has remained predominantly import-dependent. Throughout the study period, imported products accounted for the majority of registered pharmaceuticals in Sri Lanka. The share of locally manufactured products declined between 1991 and 2009, followed by a recovery in the most recent period, without altering the overall dominance of imports.

At the active-substance level, however, the share of locally manufactured substances increased substantially, rising from 11.0% (*n* = 75) in 2009 to 33.9% (*n* = 247) in 2025. This increase coincided with growth in the number of local manufacturers with at least two registered products, from 10 to 25, and with the emergence of domestic production capability for more complex dosage forms, including intravenous formulations absent from the 2009 register. Together, these developments suggest an expansion in local manufacturing capacity, despite the continued predominance of imports at the product level.

Price competition from mass-produced imported pharmaceuticals has long constrained the growth of the local pharmaceutical industry. Against this backdrop, extended government support measures, including procurement-linked buy-back guarantees, may have facilitated the recent expansion of local manufacturing activity (Government of Sri Lanka, 2024).

The composition of import sources also shifted over time. Earlier portfolios included larger shares of products from the United Kingdom, United States, Switzerland, and Germany, whereas more recent portfolios increasingly reflect sourcing from South Asian manufacturing countries. Within this shift, India’s dominant role in the registered import portfolio is likely to reflect its position as a major regional producer and exporter of generic medicines, together with geographic proximity, established trade channels, and demand for lower-cost generic products in Sri Lanka. These patterns are consistent with broader regional integration and evolving pharmaceutical trade relationships (Khan & Rauf, 2024).

Changes in sourcing patterns reinforce the importance of sustained regulatory capacity to assure medicine quality throughout the supply chain. In Sri Lanka, parliamentary oversight has documented weaknesses in storage conditions, the timely identification and communication of medicine quality failures, and quality-testing capacity (Committee on Public Accounts, 2022). The Asian Development Bank (2025) also identified limited post-registration testing, laboratory staffing constraints, and gaps in quality assurance during procurement and at the point of entry. This does not imply intrinsic differences in product quality, but rather underscores growing demands on national regulatory authorities for bioequivalence assessment, manufacturing compliance, and post-marketing surveillance (Committee on Public Accounts, 2022). In this context, registration functions not only as a gatekeeping mechanism but as a continuing instrument for safeguarding the quality and coherence of the pharmaceutical supply base.

### High within active substance proliferation and shallow registration depth

A further structural feature of the registration portfolio is the uneven distribution of product registrations across active substances. For some widely used medicines, the number of registered product variants was exceptionally high, with some active substances attracting high numbers of registrations. This pattern reflects high within active substance proliferation rather than overall concentration of the register and indicates that product registration activity is unevenly distributed across therapeutic substances.

One possible explanation for this clustering is that registration functions as a prerequisite for participation in public sector procurement. Medicines purchased in bulk through government tenders represent substantial commercial opportunities, and maintaining registration allows suppliers to remain eligible even when products are not actively marketed or widely available in the private retail sector. High registration counts for particular active substances may partly reflect the role of registration as a prerequisite for participation in public sector procurement rather than effective product availability. At very high levels, such proliferation may increase regulatory burden without substantially expanding therapeutic coverage.

In response to concerns over brand proliferation, the National Medicines Regulatory Authority introduced a registration cap in 2021, restricting new applications for active substances and strengths with fifteen or more registered products (National Medicines Regulatory Authority, 2021). The present analysis did not assess the effects of this policy, which applies to registrations per INN and strength rather than the aggregate active-substance counts examined here.

Alongside this clustering, the 2025 register reveals the opposite phenomenon: a large proportion of active substances were represented by very shallow registration depth. Of the 729 registered active substances, 380 were associated with three or fewer registered products. Among the 590 EML-listed substances, 157 had no registered products and only 168 had five or more registrations. These observations are confined to the most recent period. Low registration depth may reflect limited clinical demand, narrow therapeutic indications, or small market size; however, in a setting characterised by persistent import dependence and limited domestic production, shallow registration depth reduces redundancy within the formal supply system. This increases vulnerability to supply disruptions, heightens the risk of stockouts, and may concentrate market power among a small number of suppliers (Auditor General, 2022). Under such conditions, procurement may shift from competitive tendering toward negotiated purchasing, with implications for pricing, supply security, and regulatory leverage (Committee on Public Enterprises, 2025).

### Regulatory context and interpretation

The observed registration patterns emerged within a policy environment marked by increasing regulatory assertiveness since the early 1990s (Weerasuriya, 1993). This included active deregistration of products considered harmful or ineffective, successive efforts to articulate a national medicines policy, establishment of an independent regulatory authority, and repeated revisions of pricing and registration rules. Although this study does not assess the effects of individual policies, the cumulative regulatory context provides important background for interpreting how registration portfolios evolved under routine conditions.

## Implications for regulation and procurement

These findings have implications for pharmaceutical regulation and procurement in Sri Lanka and similar low- and middle-income settings.

First, divergence between product- and active-substance-level alignment shows that aggregate counts of registered products are a weak indicator of alignment with essential medicines priorities. Monitoring registration coverage and depth at the level of active substances would provide a more informative basis for assessing therapeutic breadth and supply vulnerabilities.

Second, the coexistence of high product proliferation for some active substances and shallow registration depth for others points to misalignment between registration and procurement incentives. Where registration primarily functions as a prerequisite for public procurement, portfolio expansion may reflect strategic positioning rather than expected supply. Greater coordination between regulatory and procurement authorities, particularly alignment of registration, renewal, and tender participation requirements, could reduce administrative burden while preserving competition.

Third, the registration cap represents a targeted response to brand proliferation. Further research should assess its effects and whether complementary measures, such as differentiated renewal criteria or linkage between registration status and market participation, are warranted.

Finally, the continued dependence on imported medicines reinforces the importance of sustained regulatory capacity across the supply chain. Product registration should be complemented by manufacturing oversight and effective post-marketing surveillance to support medicine quality throughout the product lifecycle.

## Implications for future research

Future research should link registration data with procurement, pricing, and utilisation data to clarify how portfolio structure translates into market behaviour and patient access, with particular attention to medicines with persistently low registration depth that may pose supply risks. Further work should examine how regulatory rules and procurement practices interact to shape firm registration strategies, including responses to policies such as registration caps or procurement guarantees. Comparative studies across countries with similar regulatory frameworks would help distinguish context-specific dynamics from broader structural trends.

### Strengths and limitations

A major strength of this study is the consolidation of national pharmaceutical registration data across three time points spanning more than three decades, enabling direct longitudinal comparison. In a context where registration records are not routinely archived for historical analysis, the study brings together dispersed regulatory data to examine long-term structural patterns in the registration portfolio. Use of harmonised definitions and explicit active-substance-level analysis further strengthens interpretability.

The study has limitations. Registration alignment with the EML does not necessarily indicate actual availability, procurement, or patient access, as registered products may not be marketed or continuously supplied. Alignment with essential medicines was assessed using the list in force at each study year and should therefore be interpreted relative to contemporaneous policy standards rather than a single fixed reference list. Exclusions from ATC analysis comprised combination products and records with insufficient classification information, representing 12.4% of registrations in 2009 and 3.8% in 2025. This difference may affect comparisons of therapeutic composition across years. The analysis is descriptive and does not assess causal drivers.

## Conclusions

This study provides a longitudinal perspective on pharmaceutical registration in Sri Lanka, updating and extending the 1993 assessment of essential medicines alignment under routine policy conditions. Essential medicines continue to shape the registration portfolio, but the structure of that alignment has changed substantially over time.

The shift from high product-level alignment toward broader active-substance-level coverage reflects evolving market organisation, procurement incentives, and regulatory practice. At the same time, the coexistence of intense product proliferation for some active substances and limited registration depth for others reveals structural tensions with implications for regulatory workload, supply security, and market power.

Overall, the findings indicate that pharmaceutical registration portfolios provide an important lens through which to examine therapeutic coverage, registration concentration, and potential supply vulnerability within import-dependent pharmaceutical systems. Monitoring registration depth at the level of active substances may help support more coherent regulatory and supply planning.

## Data Availability

The supporting analytical datasets generated for this study are available through the University of Sydney’s Sydney eScholarship repository: 10.25910/bb1b-0h30.
